# Early Fruit Development Regulation-Related Genes Concordantly Expressed with TCP Transcription Factors in Tomato (*Solanum lycopersicum*)

**DOI:** 10.3390/cimb45030153

**Published:** 2023-03-13

**Authors:** Sherif Edris, Aala A. Abulfaraj, Rania M. Makki, Salah Abo-Aba, Mardi M. Algandaby, Jamal Sabir, Robert K. Jansen, Fotouh M. El Domyati, Ahmed Bahieldin

**Affiliations:** 1 Department of Biological Sciences, Faculty of Sciences, King Abdulaziz University, Jeddah 21589, Saudi Arabia; 2 Princess Al-Jawhara Al-Brahim Centre of Excellence in Research of Hereditary Disorders (PACER-HD), Faculty of Medicine, King Abdulaziz University, Jeddah 21589, Saudi Arabia; 3 Department of Genetics, Faculty of Agriculture, Ain Shams University, Cairo 11241, Egypt; 4 R&D Department, Al Borg Diagnostics, Jeddah 23514, Saudi Arabia; 5 Biological Sciences Department, College of Science & Arts, King Abdulaziz University, Rabigh 21911, Saudi Arabia; 6 National Research Centre, Department of Microbial Genetics, Genetic Engineering and Biotechnology Division, Giza 12622, Egypt; 7 Department of Integrative Biology, University of Texas at Austin, Austin, TX 78712, USA

**Keywords:** Chico III, concordantly expressed genes, gene regulation RNAseq, transcription factors

## Abstract

The tomato (*Solanum lycopersicum* L.) is considered one of the most important vegetable crops globally, both agronomically and economically; however, its fruit development regulation network is still unclear. The transcription factors serve as master regulators, activating many genes and/or metabolic pathways throughout the entire plant life cycle. In this study, we identified the transcription factors that are coordinated with *TCP* gene family regulation in early fruit development by making use of the high-throughput sequencing of RNA (RNAseq) technique. A total of 23 *TCP*-encoding genes were found to be regulated at various stages during the growth of the fruit. The expression patterns of five *TCPs* were consistent with those of other transcription factors and genes. There are two unique subgroups of this larger family: class I and class II TCPs. Others were directly associated with the growth and/or ripening of fruit, while others were involved in the production of the hormone auxin. Moreover, it was discovered that TCP18 had an expression pattern that was similar to that of the ethylene-responsive transcription factor 4 (ERF4). Tomato fruit set and overall development are under the direction of a gene called auxin response factor 5 (ARF5). TCP15 revealed an expression that was in sync with this gene. This study provides insight into the potential processes that help in acquiring superior fruit qualities by accelerating fruit growth and ripening.

## 1. Introduction

Tomato (*Solanum lycopersicum* L.) is among the most important vegetable crops worldwide in terms of economics and agronomy [1,2]. The development of the fertilized tomato fruit ends at the red ripe stages [3,4]. Many transcription factors participate in regulating the course of fruit development. Some act as master switches for provoking several genes and/or metabolic pathways involved in all stages of plant development [5,6].

*TCP* is a transcription factor (TF) gene family involved in biological processes such as senescence, circadian rhythm, and hormone signaling [7,8]. *TCPs* were discovered in 1999 and named after three gene family members encoding TEOSINTE BRANCHED 1 (TB1) from maize, CYCLOIDEA (CYC) from *Antirrhinum majus*, and PROLIFERATING CELL FACTORS 1 and 2 (PCF1 and PCF2) from rice [7], all of which are characterized by the occurrence of the TCP domain, a motif comprising a 59-residue-long non-canonical basic helix–loop–helix (bHLH) structure 1 [9]. In tomato, 30 TCPs have been detected, of which 13 are class I TCPs, namely, *TCP1-11*, *TCP23*, and *TCP27*. Class II in tomato consists of 11 TCPs, namely, *TCP1-6*, *TCP10*, *TCP24*, and *TCP28-30* for the CIN subfamily, and 6 TCPs, namely, *TCP7-9*, *TCP22*, *TCP25*, and *TCP26*, for subfamily CYC/TB1. The two classes differ by a four-amino-acid deletion in the basic region of the TCP domain of class I relative to class II proteins [2].

Examples of class I include *TCP14* which regulates embryonic growth potential in the Arabidopsis seeds [10] and *TCP15* which controls the internode length [11]. The *TCP20* gene functions in several developmental processes, e.g., growth processes [12] and leaf senescence [13], while the *TCP16* gene is predominantly expressed in developing microspores [14]. In conjunction with DICHOTOMA (DICH), the CYC class II gene is required for the dorsoventral asymmetry of the flower in *Antirrhinum* [15]. *TCP1*, a CYC/DICH homolog, is linked to growth [16]. The *TB1* gene prevents the outgrowth of buds at lower nodes while promoting the formation of female inflorescences at higher nodes [17]. Two homologs of this gene, e.g., *TCP18* and *TCP12* are expressed in axillary buds, thus preventing branching [18]. The *TCP2* gene also affects plant architecture in *Antirrhinum* [19] and tomato [20], while *TCP4* and *TCP21* genes are required for petal growth and development [21] and circadian clock regulation in Arabidopsis [22]. Moreover, there is a dominant negative variant of TCP3 that results in shorter siliques with a wrinkled surface [23]. In general, *TCP2*, *TCP3*, *TCP4*, *TCP10*, and *TCP24* are implicated in regulating leaf morphogenesis [23,24,25]

The genes involved in the ripening process have been identified via extensive research into the development and maturation of tomato fruit. We now know the names and characteristics of these genes [26,27], but there is still a knowledge gap in understanding the regulation at the early fruit development stage; so, in the present study, an RNA-Seq dataset of gene expression in tomato was retrieved to validate *TCP* expression patterns throughout the early fruit development stage. Moreover, we look at how well TCP lines up with genes that are active in the first stages of tomato fruit development using the Chico III cultivar (*Solanum lycopersicum*).

## 2. Materials and Methods

Tomato plants cv. Chico III were cultivated in a greenhouse at 16/8 h and 25 °C/18 °C day/night cycle, 80% humidity, and 250 mol per second and square meter (m^−2^ s^−1^) light intensity, with frequent applications of fertilizer. In order to determine when exactly the fruits were ready for picking, we tagged flowers on their anthesis day.

The flower samples were taken at six growth stages, i.e., 0 days after pollination (DAP), 3 DAP, 5 DAP, 7 DAP, 9 DAP, and 12 DAP, as described in [28]. RNAs were isolated at different time points and sent to Beijing Genomics Institute (BGI) in China for deep sequencing. RNA-Seq raw reads were placed in Sequence Read Archive (SRA) database (http://www.ncbi.nlm.nih.gov/Traces/sra/) (accessed on 2 January 2023) under the accession number SUB1151548, and bioinformatics analysis was performed as described in [28,29]. Clusters with TCP transcription factors and concordantly expressed genes were selected for further analysis. Quantitative reverse transcription polymerase Chain Reaction (qRT-PCR) was performed to validate RNA-Seq data of selected genes, as described in [28]. qRT-PCR primer pairs (18–20 bp) were designed using Premier 5.0 software (Premier Biosoft International, Palo Alto, CA, USA). The glyceraldehyde-3-phosphate dehydrogenase or GAPDH (accession no. U93208) from *S. lycopersicum* was used as the housekeeping gene.

## 3. Results and Discussion

**Figure 1:**
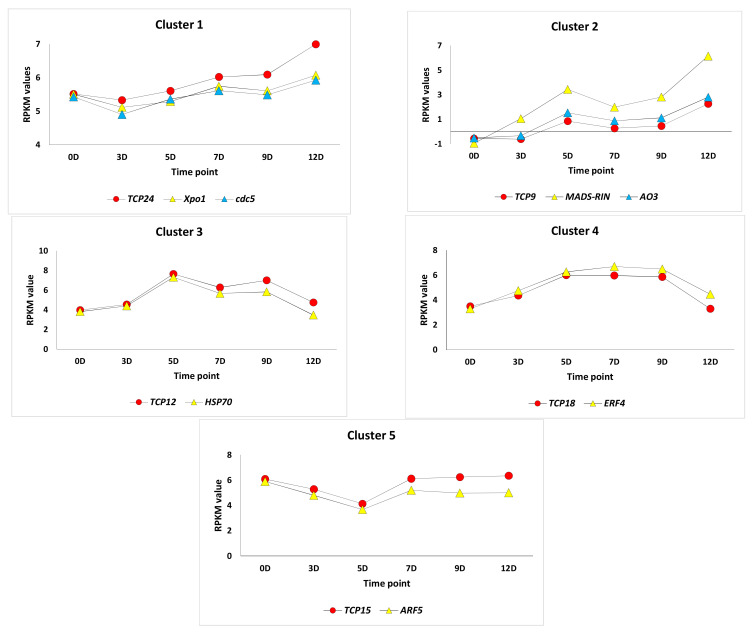
The concordant expression between *TCPs* and other genes related to fruit development across the time of early fruit development (0-12D) in tomato cultivar Chico III. Clusters were upregulated at five (cluster 1), four (cluster 2), three (cluster 3), two (cluster 4), and one (cluster 5) time point.

The data shown in Table S1 indicate the regulation of 23 out of the 30 genes encoding TCPs of the two classes during early fruit development in tomato cultivar Chico III. They are *TCP11-21* and *TCP23* of class I; *TCP1*, *TCP3-6*, *TCP10*, and *TCP24* of the CIN subfamily; and *TCP7-9* and *TCP22* of CYC/TB1. Five of these TCP genes showed concordant expression with other transcription factors and genes (Figure 1). The concordantly expressed TF//TF or TF//gene include *TCP24*//*Xpo1*//*cdc5* (cluster 1), *TCP9*//*MADS-RIN*//*AO3* (cluster 2), *TCP12*//*HSP70* (cluster 3), *TCP18*//*ERF4* (cluster 4), and *TCP15*//*ARF5* (cluster 5). Considering the six time points (0, 3, 5, 7, 9, and 12D) to describe the five stages of early fruit development, cluster 1 showed regulation in the five stages. In contrast, clusters 2, 3, 4, and 5 showed regulation in four, three, two, and one stage, respectively (Figure 1). The five concordantly expressed *TCP* genes exist in class I (*TCP12*, *TCP15*, and *TCP18*), CIN (*TCP24*), and CYC/TB1 (*TCP9*).

TCP transcription factors are very dynamic during early growth stages, e.g., seed germination, cell cycle regulation, etc., as well as during later stages, e.g., circadian rhythm, hormone signaling, floral organ morphogenesis, and pollen/leaf development up to senescence [8,13,30]. *TCP* genes of class II have been well defined in Arabidopsis, tobacco, and tomato [9,31]. However, little is known about the function of class I proteins. Among the *TCP* genes of this class, *TCP12* (*BRC2*) and *TCP18* (*BRC1*) played a general role in controlling plant architecture and were expressed explicitly in tomato fruit, suggesting their participation in fruit development and/or ripening, while the *TCP15* gene was shown to participate in the auxin pathway in Arabidopsis [30]. *TCP14* and *TCP15* genes have overlapping functions in regulating internode cell proliferation, branching, and meristem development in Arabidopsis [11,32,33]. Overall, overlapping functions exist between the class I and II proteins in both cell growth and division. Recent reports indicate that genes encoding *TCP12*, *TCP18*, and *TCP15* are regulated by RIN (RIPENING INHIBITOR), CNR (COLORLESS NON-RIPENING), and SlAP2a (APETALA2a) proteins. The latter proteins represent transcription factors with key roles in ripening. *TCP9* (*BRC1A*) and *TCP24* contain the R domain C terminal of the TCP domain [13].

**Figure 2:**
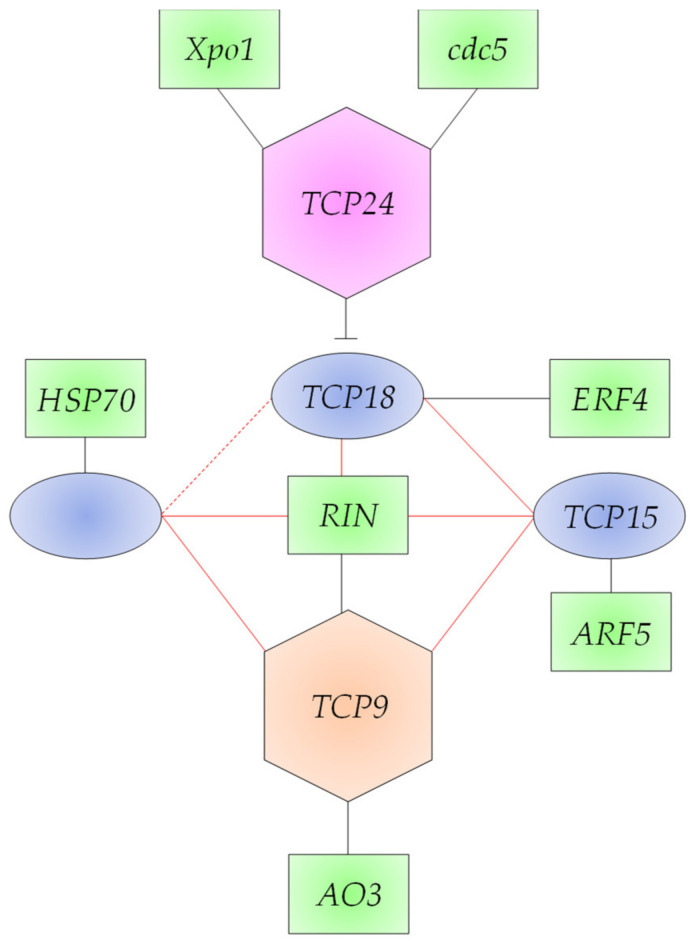
Gene networking generated from transcriptome-based cluster analysis for the *TCP* genes (in red, blue, or lilac) concordantly expressed with other genes (in green) in tomato cultivar Chico III. Black lines indicate interactions based on the transcriptomic results of the present study, while red lines indicate interactions based on the yeast one-hybrid results of the Parapunov [9]. Solid lines indicate direct connections, while the dashed line indicates intermediate connection(s).

**Figure 3:**
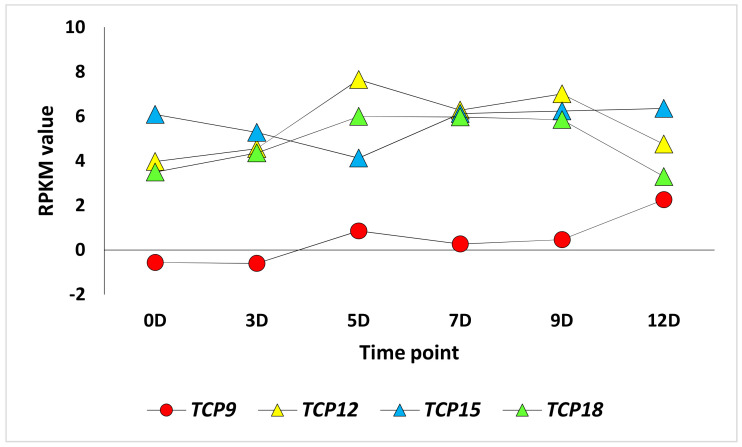
Expression profiles of the four *TPC* genes, namely, *TPC9*, *TPC12*, *TPC15*, and *TPC18* during early fruit development stages in tomato cultivar Chico III.

It was also reported that TCPs in tomato regulate one another and/or crosstalk to regulate downstream genes involved in fruit development and ripening. Through the yeast one-hybrid assay, it was proven that *TCP9* binds to promoters of *TCP12*, *TCP15*, and *TCP18* genes to drive their expression. Then, the expression of the three crucial TCPs (*TCP12*, *TCP15*, and *TCP18*) occurs downstream of the expression of *TCP9*. In the present study, the latter results seem to be true for *TCP12* and *TCP18* as they were concordantly expressed with *TCP9* during the first four time points, while opposite expression patterns were found when comparing *TCP9* and *TCP15* (Figure 2). Expectedly, the *TCP24* gene showed no concordant expression with *TCP12*, *TCP15*, or *TCP18* genes (Figure 3).

**Figure 4:**
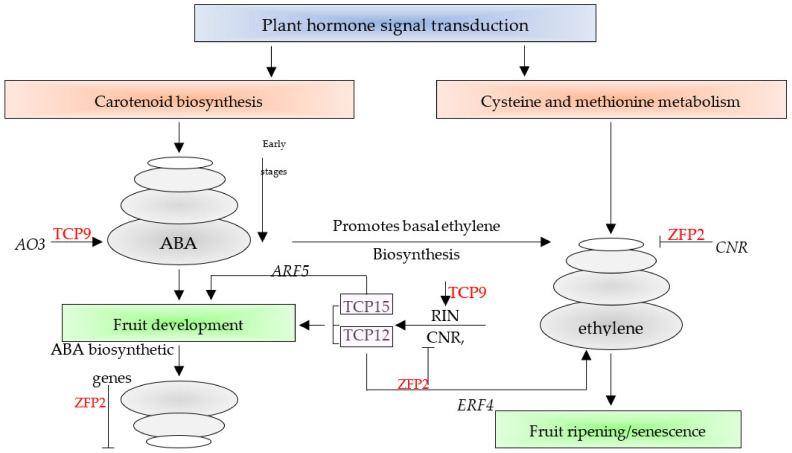
Regulation of fruit development and ripening is mutually driven by *TCP9* and *ZFP2* in tomato, respectively. *TCP9* possibly drives the expression of the *AO3* gene towards ABA biosynthesis, while *ZFP2* drives the suppression of the *CNR* gene towards ethylene production. Modulation between ABA and ethylene is also driven by *ZFP2*. Regulation of genes encoding *TCP12*, *TCP15*, and *TCP18* is also shown, along with two concordantly expressed genes, namely, *ERF4* and *ARF5*.

The results of cluster 1 indicate concordant expression of *TCP24*, *XpoI*, and *cdc5* genes (Figure 1 and Figure 4). *TCP24* participates in the control of morphogenesis of shoot organs in Arabidopsis by negatively regulating the expression of a boundary-specific gene, e.g., CUC, through miRNA induction (e.g., miR164). This *TCP*, along with Armadillo BTB Arabidopsis protein 1 (*ABAP1*), negatively participates in the leaf cell proliferation [34] by binding specifically to the promoters *AtCDT1a* and *AtCDT1b*. The latter proteins are members of the prereplication complex and plastid division machinery [35]. The *Xpo1* gene encodes Exportin 1, a nuclear export receptor for proteins carrying leucine-rich nuclear export signals (NESs) in Arabidopsis [36,37] and tomato [38]. The gene is required during gametophyte development [39] and participates in heat-induced oxidative stress basal resistance [40]. The *cdc5* gene in Arabidopsis is a component of the MAC complex essential for plant innate immunity and participates in mRNA splicing and cell cycle control [41,42]. Knockdown of this gene can accelerate cell death in the plant [43]. The interesting upregulation pattern of expression can be explained by the initiation of programmed cell death as early as the fruit development stages.

It is known that *TCP9* participates in delayed leaf senescence and root development [13]. Chen et al. [44] revealed that *TCP9* and *TCP15* participate in even later stages as they are expressed in archespores, pollen mother cells, ovule primordia, and megaspore mother cells. Interestingly, the expression pattern of *TCP9* is almost opposite to that of *TCP15* in the present study, as they are mutually upregulated during early fruit development stages. *TCP9* was proven to interact with *SPOROCYTELESS* or *SPL*, a gene controlling germline formation in the plant. This result directly links *TCP9* with early fruit development. The results in Figure 1 and Figure 3 indicate the indirect link of *TCP9* with fruit ripening as it seems that this TF drives expression of another TF, namely, MADS-ripening inhibitor or MADS-RIN, that functions in fruit development [45] and induces fruit ripening in tomato [46]. The nomenclature of MADS came from an acronym referring to four founding members, namely, MCM1, AGAMOUS, DEFICIENS, and SRF [47]. The results of cluster 2 (Figure 1 and Figure 4) indicate a possible indirect action of *TCP9* in fruit development and ripening that occurred by driving the *aldehyde oxidase 3* (*AO3*) gene. The enzyme encoded by the *AO3* gene catalyzes the oxidation of the abscisic aldehyde to ABA [47]. ABA was proven to participate in fruit development and ripening processes [48]. The two latter processes occur oppositely but simultaneously in the plant hormone signal transduction pathways. ABA synthesized in the carotenoid biosynthesis pathway is responsible for the fruit development stage and modulates the fruit ripening stage, while ethylene synthesized in cysteine and methionine metabolism pathways participates in fruit ripening only. The larger the time between fruit development and ripening processes, the larger the tomato fruit size and yield. Two TFs, namely, *ZFP2* [48] and *TCP9*, seem to act mutually during these two processes. Biosynthesis of ABA by *AO3*, possibly driven by *TCP9*, seems to favor fruit development, while modulation between ABA and ethylene, driven by ZFP2 (zinc finger protein 2), appears to favor ripening (Figure 4). Suppression of the ABA biosynthetic and *COLORLESS NON-RIPENING* (*CNR*) genes, driven by ZFP2, takes place right after the fruit development stage to help promote the basal level of ethylene biosynthesis.

*TCP12*, *TCP15*, and *TCP18* were recently reported to be regulated by the ripening regulators *RIN*, *CNR*, and *AP2a* in tomato ([9] and Figure 4 of the present study). The latter protein, namely, adaptor protein 2, is involved in floral organ development [49]. A decrease in the three proteins results in the switch from fruit development to ripening. In the present study, RIN was shown to be regulated by *TCP9*, while no information is available to confirm the connection of the TCPs, other than *TCP12*, *TCP15*, and *TCP18*, with the other two proteins. The results of cluster 3 (Figure 1 and Figure 4) indicate that a gene encoding one of the *HSP70* genes seems to be regulated by *TCP12*. *HSP70* genes are involved in tomato fruit response to the fungus *R. nigricans.* This study casts light on the possible role of *TCP12* in response to biotic and abiotic stresses [50]. As the plants in this study showed no signs of fungal infection in the greenhouse, HSP70 might be accumulated as a precaution for possible heat stress during the critical stages of fruit development taking place in the summer. The results of the *TCP18* expression pattern in cluster 4 (Figure 1 and Figure 4) indicate its possible regulation of ethylene-responsive transcription factor 4 or *ERF4* gene. The latter TF is among the downstream components of ethylene signaling that regulate the expression of ethylene-responsive genes [51].

Interestingly, the over-expression of one ERF member (*ERF.B3*) resulted in a dramatic delay in fruit ripening in tomato. These data suggest pleiotropic alterations caused by *ERF* genes during fruit maturation and ripening [51]. Therefore, we speculate that *TCP15* might be a possible modulator of fruit development by inducing the accumulation of ABA and fruit ripening by inducing the proliferation of ethylene. The results of the *TCP15* gene in cluster 5 (Figure 1 and Figure 4) indicated the concordant expression with auxin response factor 5 or the *ARF5* gene. The *ARF* gene family functions in the regulation of plant development processes. ARF5 was most recently proven to regulate tomato fruit set and development via the mediation of auxin and gibberellin signaling [50].

In conclusion, the present study casts light on the mechanisms that can promote fruit development at the expense of fruit ripening to increase tomato fruit yield and storage time.

## 4. Conclusions

In conclusion, *TCP18* and *TCP15* expressed ethylene-responsive transcription factor 4 (*ERF4*), and auxin response factor 5 (*ARF5*) affect tomato fruit set and development. This study illuminates processes that may stimulate tomato fruit development at the expense of ripening to increase production and storage time. These mechanisms aid tomato fruit growth and ripening.

## Data Availability

Raw reads were placed in the Sequence Read Archive (SRA) database (http://www.ncbi.nlm.nih.gov/Traces/sra/ (accessed on 2 January 2023)) under the accession number SUB1151548.

## Supplementary Materials

The following are available online at https://www.mdpi.com/article/10.3390/cimb45030153/s1, Table S1: Expression patterns of genes regulated during early stages of fruit ripening, Table S2: Expression patterns of genes regulated during early stages of fruit ripening. Regulated TCP genes are highlighted in yellow color.
